# Nonfat milk attenuates acute hyperglycemia in individuals with android obesity: A randomized control trial

**DOI:** 10.1002/fsn3.767

**Published:** 2018-09-12

**Authors:** Miriam P. Leary, Stephen J. Roy, Jisok Lim, Wonil Park, Rodrigo Ferrari, Jared Eaves, Daniel R. Machin, Hirofumi Tanaka

**Affiliations:** ^1^ Cardiovascular Aging Research Laboratory Department of Kinesiology and Health Education The University of Texas at Austin Austin Texas

**Keywords:** abdominal obesity, dairy, flow‐mediated dilation, oral glucose tolerance test

## Abstract

**Background:**

Elevated android body fat increases the risk of developing cardiometabolic diseases. Postprandial hyperglycemia contributes to the proatherogenic metabolic state evident in android adiposity. Due to the insulinotropic effect of milk‐derived proteins, postprandial hyperglycemia has been shown to be reduced with the addition of dairy products. The purpose of this study was to determine whether one serving of nonfat milk added to an oral glucose tolerance test (OGTT) could attenuate postprandial hyperglycemia in individuals with elevated android adiposity and whether these improvements would be associated with metabolic and/or peripheral hemodynamic effects.

**Methods:**

In this placebo‐controlled, randomized, crossover experimental study, 29 overweight/obese adults (26 ± 1 year) consumed an OGTT beverage (75 g glucose) combined with either nonfat milk (227 g) or a placebo control (12 g lactose + 8 g protein + 207 g water) that was matched for both carbohydrate and protein quantities.

**Results:**

In the whole sample, blood glucose and insulin concentrations increased over time in both trials with no significant differences between trials. Relative increases in peak blood glucose response were significantly related to android body fat (*p* < 0.05). The subjects in the highest tertiles of android body fat displayed attenuated hyperglycemic responses as well as improvements in flow‐mediated dilation (FMD) after milk intake.

**Conclusions:**

A single serving of nonfat milk may attenuate acute hyperglycemia in individuals with elevated android body fat offering a simple and convenient option for managing elevations in blood glucose.

## 1 INTRODUCTION

Obesity has long been accepted as a clinical risk factor for cardiovascular and other chronic diseases (Chan, Rimm, Colditz, Stampfer, & Willett, 1994; Ortega, Lavie, & Blair, 2016). In particular, android obesity (defined as central or abdominal obesity with excess distribution of body fat in the abdomen) is associated with greater risk of developing cardiometabolic diseases than other adipose storage patterns (Pinnick et al., 2014) as it exacerbates metabolic disturbance (Hanley et al., 2004). The visceral adipose tissue that coexists with android obesity is associated with insulin resistance as well as reduced glucose disposal and oxidation (Ibrahim, 2010). Traditionally, fasting plasma glucose has been used as a clinical determinant of CVD risk. However, as individuals spend the majority of their time in a postprandial state, acute responses to plasma glucose following an oral glucose tolerance test (OGTT) are better predictors of relative CVD risk (Donahue, Abbott, Reed, & Yano, 1987). Indeed, increases in postprandial hyperglycemia following a high carbohydrate meal contribute importantly to a proatherogenic metabolic state (Cavalot et al., 2006) in part through attenuating endothelium‐dependent vasodilation (Kawano et al., 1999; Williams et al., 1998).

Dairy consumption is inversely associated with the risk of cardiometabolic diseases (Elwood, Pickering, Hughes, Fehily, & Ness, 2004; Mennen et al., 2000; Ostman, Liljeberg Elmstahl, & Bjorck, 2001; von Post‐Skagegard, Vessby, & Karlstrom, 2006; Qin et al., 2015). Importantly, the beneficial effects of dairy on metabolic and clinical parameters are evident in individuals with elevated risk of CVD (Elwood, Pickering, Givens, & Gallacher, 2010; Pereira et al., 2002). However, there appears to be a distinct “floor effect” associated with dairy intake, in which little to no changes were observed in relatively healthy individuals with normal or low baseline levels of cardiovascular risk factors (Poppitt et al., 2002). The complete mechanism underlying the association between dairy intake and cardiometabolic diseases is not clear. However, dairy products may attenuate the risk of metabolic dysfunction through reductions in postprandial glycemia when milk and milk products are consumed with a meal (Fekete, Givens, & Lovegrove, 2016). Recent research has demonstrated favorable effects in glycemic management when dairy products are combined with high‐carbohydrate meals (Fekete et al., 2016; Panahi, El Khoury, Luhovyy, Goff, & Anderson, 2013; Panahi et al., 2013). The majority of relevant findings in this area attribute attenuated hyperglycemia to the insulinotropic properties of dairy proteins (Akhavan, Luhovyy, Brown, Cho, & Anderson, 2010; Fekete et al., 2016; Floyd, Fajans, Conn, Knopf, & Rull, 1966; Jakubowicz et al., 2014; Manders et al., 2014; Schmid, Schusdziarra, Schulte‐Frohlinde, Maier, & Classen, 1989; Schmid et al., 1992). In fact, milk‐derived proteins elicit dose‐dependent decreases in postprandial hyperglycemia when consumed with a high‐carbohydrate meal (Akhavan et al., 2010). However, no study has yet investigated whether milk can attenuate postprandial hyperglycemia independent of its protein quantity. Additionally, it is unknown if the beneficial effects of milk are mediated by changes in peripheral vasodilation and hemodynamics.

Therefore, the purpose of this study was to determine whether a serving of nonfat milk could attenuate postprandial hyperglycemia independent of its protein quantity. As android obesity is associated with poorer metabolic health, we sought to determine the influence of android body fat on the milk's ability to reduce postprandial hyperglycemia. Based on the observation that the effects of dairy intake do not appear to manifest in relatively healthy individuals (Poppitt et al., 2002), we hypothesized that milk would attenuate an acute hyperglycemic load compared with a macronutrient, caloric control in individuals with the greatest level of adiposity. Moreover, we posited that these postulated improvements would be associated with peripheral vasodilation.

## 2 METHODS

### 2.1 Study population

A total of 29 adults with a mean (±*SEM*) age of 26 ± 10 year were studied. Participants were recruited via advertisements and fliers from the local community of Austin, Texas, between April 2013 and December 2015. Inclusion criteria were as follows: apparently healthy, sedentary (physical activity <3 day/week), overweight or obese (BMI ≥ 25.0 kg/m^2^), nonsmokers, no overt signs of chronic diseases on physical examination or medical health history, normal blood chemistry as assessed by fasting glucose and lipid panel, no cardiovascular‐acting medications, and no pregnancy. Participants who were lactating or presented with dairy allergies, lactose intolerance, or alcohol abuse were excluded from the study. Participants were instructed to maintain their normal routine diet and exercise habits for the duration of the study. After being informed about the study verbally and in writing, all participants gave their informed consent. The study was conducted in accordance with the Declaration of Helsinki, and all procedures were reviewed and approved by the Internal Review Board at The University of Texas at Austin. This study was registered on clinicaltrials.gov (#NCT02894112).

### 2.2 Study design

A placebo‐controlled, randomized, crossover experimental design was used for the present investigation. Order of interventions was determined by a random sequence generator and administered by the lead investigator (MPL). Each participant underwent both nonfat milk and placebo treatments with an OGTT. Each treatment was preceded by two consecutive days of strict diet and physical activity controls. Treatments were separated by a washout period of at least 1 week during which time subjects resumed normal dietary and physical activity patterns. Though initially designed to include a third arm (a carbohydrate control trial), it was deemed unnecessary in answering the present question as the comparison it would have allowed has already been well established (Akhavan et al., 2010; Petersen et al., 2009).

### 2.3 Experimental protocol

All testing visits and laboratory work were conducted in a quiet, temperature‐controlled room in the Cardiovascular Aging Research Laboratory at the University of Texas at Austin. During the screening visit, body composition was assessed using dual‐energy X‐ray absorptiometry (DXA; see below). Using lean body mass assessed by DXA, daily caloric requirements were calculated for the standardized meals (Medicine ACoS, 2013). Nonperishable, travel‐friendly, standardized meals were matched for energy content (isocaloric; 60% carbohydrate, 15% protein, and 25% fat) and were provided to participants to consume on Days 1–2. The participants were provided with a dietary record log and were instructed to consume the same meals and record the timing of the meals on both days to better replicate the dietary controls prior to the second treatment. Alcohol and caffeine intake were prohibited starting in the evening before Day 1. In addition to the diet controls, the participants were instructed to maintain their normal daily activity but refrain from both formal and recreational exercises. To confirm this, participants were provided with and required to wear pedometers during the waking hours of Days 1–2. Participants recorded food intake compliance and daily step counts on a record log that was submitted to investigators the morning of Day 3. The food record log was analyzed by a registered dietician using Nutrition Pro software (Axxya Systems, Stafford, TX) which is based on a comprehensive food knowledge database with over 51,000 foods and ingredients.

Following these two control days, participants reported to the laboratory on the morning of Day 3 after at least a 12‐hr fast for resting measures followed by one of two treatments. An intravenous catheter was inserted into the antecubital vein, and fasting blood samples were collected. Upon conclusion of the resting measures, participants consumed a fruit punch flavored, standard OGTT beverage (100 g glucose) combined and mixed with either: 8 oz of nonfat milk (227 g) or 8 oz of the control drink (12 g lactose + 8 g whey protein + 207 g water). The placebo control drink was identical to nonfat milk in macronutrient and calorie contents as well as color. Participants were instructed to consume the test beverages within 5 min. After ingestion of test solutions, the participants remained in the supine position during the 2‐hr postprandial period in the quiet, temperature‐controlled laboratory.

### 2.4 Measurements

The primary outcome for this study was blood glucose concentrations. Secondary measures include other metabolic markers including insulin, glucagon, gastric inhibitory polypeptide (GIP) concentrations, as well as hemodynamic measures including flow‐mediated dilation (FMD), femoral artery flow, and conductance, blood pressure, and heart rate.

Body composition and android body fat were estimated noninvasively using the total body scan by DXA (GE Lunar, Chicago, IL) (Mazess, Barden, Bisek, & Hanson, 1990). For measuring android fat, a region of interest was automatically defined by the software, in which the caudal limit was placed at the top of the iliac crest and its height set to 20% of the distance from the top of the iliac crest to the base of the skull to define its cephalad limit (Stults‐Kolehmainen et al., 2013). During each treatment, blood samples were collected at baseline as well as 10, 20, 30, 45, 60, 90, and 120 min during the postprandial period. These samples were later analyzed for plasma glucose, insulin, glucagon, and GIP concentrations. Commercially available assay kits were used to determine plasma concentrations of glucose (Point Scientific, Canton, MI), GIP (RayBio, Norcross, GA), and insulin (Mercodia, Uppsala, Sweden). A commercially available radioimmunoassay kit was used to assess glucagon (EMD Millipore, Darmstadt, Germany) (Clevenger, Parker Jones, Tanaka, Seals, & DeSouza, 2002). The net incremental area under the curve (iAUC) for plasma glucose and insulin was calculated using the trapezoidal method (Clevenger et al., 2002) as it was shown to be more strongly correlated with glycemic rise than total AUC (Le Floch, Escuyer, Baudin, Baudon, & Perlemuter, 1990).

Vascular function was measured at baseline, 30 min, and end of the postprandial period (120 min). To assess vascular endothelium‐dependent vasodilation, FMD was assessed as previously described (Harrison, Parkhurst, Tarumi, Lin, & Tanaka, 2011). Briefly, brachial artery diameters and blood flow velocity were measured from images derived from an ultrasound machine (iE33, Philips Medical, Bothell, WA) equipped with a high‐resolution linear‐array transducer. A longitudinal image of the brachial artery was acquired 5–10 cm proximal to the antecubital fossa. A blood pressure cuff, placed on the forearm 3–5 cm distal to the antecubital fossa, was inflated to 50 mmHg above resting systolic blood pressure or a maximum of 200 mmHg for 5 min. After cuff deflation, ultrasound‐derived measurements of the brachial artery diameters and blood velocity were taken for 3 min. FMD was calculated as a percent increase in brachial artery diameter at the postblood flow occlusion compared with the preblood flow occlusion.

Blood flow and vascular conductance were measured in the common femoral artery using the ultrasound machine (iE33, Philips Medical, Bothell, WA) as previously described (Anton et al., 2006a; Dinenno, Jones, Seals, & Tanaka, 1999). To minimize turbulence from the bifurcation, the measurements were performed below the inguinal ligament, approximately 2–3 cm above its bifurcation into the profundus and superficial branch. Mean blood velocity measurements were performed with the insonation angle <60°. Blood flow was calculated from the following formula: (mean blood velocity) × (circular area) × 6 × 10^4^. The data were analyzed by the same investigator, who was blinded to the identity of the participant and the treatment. Femoral artery vascular conductance was calculated as femoral blood flow/mean arterial pressure.

### 2.5 Power calculations

All power calculations were performed using PASS software (NCSS Statistical Software, Kaysville, UT). Based on means, *SD*, and anticipated effect sizes from previous studies, sample sizes were determined for group differences in glucose responses to dietary (primarily dairy) interventions (Gannon, Nuttall, Lane, & Burmeister, 1992; Liljeberg Elmstahl & Bjorck, 2001; Nilsson, Stenberg, Frid, Holst, & Bjorck, 2004; Ostman et al., 2001; von Post‐Skagegard et al., 2006). The power calculation for femoral blood flow and vascular conductance was performed on our previous published work because there are no published group data for these variables (Anton et al., 2006b; Dinenno, Tanaka, Stauffer, & Seals, 2001; Dinenno et al., 2001). The magnitude of group differences ranged from 15% (arterial blood pressure) to 45% (femoral vascular resistance), representing physiologically meaningful differences. The power calculations using sample sizes of 30 subjects per group with α (the probability of a type I error) chosen to be 0.05 arrived at power values of 0.85 to 0.99, which are equivalent to a probability of less than 0.15 of committing a type II error. Therefore, the selected sample size of *30 subjects* was deemed sufficient to show significant differences (if present).

### 2.6 Statistical analyses

Two‐way (treatment × time) ANOVA with repeated measures were used to analyze the effects of treatment solutions. Tukey least significant difference test was used for all post hoc comparisons. iAUC for plasma glucose, insulin, glucagon, and GIP concentrations was calculated. Linear regression analyses were performed to determine associations between insulin and glucose iAUCs for both trials at 30 and 120 min. As well as the relation between relative increases in peak blood glucose concentration [(peak blood glucose – baseline blood glucose)/baseline blood glucose] and android body fat levels. Changes in FMD were compared between conditions with paired t tests. All data were expressed as means ± *SEM*.

## 3 RESULTS

Selected participant characteristics are reported in Table 1. Despite being overweight and obese, participants had normal blood pressure and fasting blood glucose, lipid, and lipoprotein concentrations. Step counts, as assessed by the pedometer, did not differ throughout the testing period between the two trials (*p* > 0.05). There were no differences between sexes/genders identified for any primary outcome variable.

**Table 1:** Selected participant characteristics

Variable	Mean ± *SEM*
*n*	29
Age (year)	26 ± 1
Men/women (*n*)	17/12
Height (cm)	172 ± 1
Body weight (kg)	92.7 ± 2.0
BMI (kg/m^2^)	31.6 ± 0.9
Total body fat (%)	39 ± 2
Android body fat (%)	48 ± 2
Systolic BP (mmHg)	116 ± 2
Diastolic BP (mmHg)	77 ± 2
Total cholesterol (mg/dl)	180 ± 8
LDL cholesterol (mg/dl)	108 ± 7
HDL cholesterol (mg/dl)	48 ± 7
Triglycerides (mg/dl)	107 ± 17
Blood glucose (mg/dl)	92 ± 2

Notes. BMI: body mass index; BP: blood pressure; LDL: low‐density lipoprotein; HDL: high‐density lipoprotein.

As shown in Table 2, blood glucose and insulin concentrations increased over time in both the milk and the placebo control drink trials. There were no significant differences (all *p* > 0.05) in glucose concentrations, insulin concentrations, glucose iAUC, or insulin iAUC between the nonfat milk and placebo control over the two‐hour postprandial period (at 120 min). Glucose iAUC was related to insulin iAUC (*R* ^2 ^= 0.40, *p* < 0.05) for 30 min, and the association was stronger when the entire 120 min were included (*r* ^2 ^= 0.71, *p* < 0.05). There were no significant differences in plasma glucagon or GIP responses between the test beverages over the two‐hour postprandial period. Similarly, there was no difference between the test beverages for glucagon or GIP iAUC (data not reported). There were no significant correlations between age and glycemic responses.

**Table 2:** Changes in metabolic hormone concentrations throughout the oral glucose tolerance test (OGTT) with nonfat milk or the control drink

Measure	Trial	OGTT							
		Baseline	10	20	30	45	60	90	120
Glucose (mg/dl)	Milk	84 ± 2	95 ± 3^a^	116 ± 4^ab^	123 ± 4abc	124 ± 5abc	119 ± 5^ab^	111 ± 5abdef	101 ± 4acdefg
	Control	86 ± 3	100 ± 4^a^	122 ± 5^ab^	123 ± 6^ab^	127 ± 8^ab^	120 ± 7^ab^	111 ± 6abcdef	102 ± 5acdefg
Insulin (pmol/l)	Milk	66 ± 6	329 ± 41^a^	647 ± 64^ab^	700 ± 74^ab^	710 ± 71abc	705 ± 76abc	675 ± 78^ab^	595 ± 91abdefg
	Control	66 ± 6	395 ± 42^a^	687 ± 66^a^	729 ± 57^a^	695 ± 81^a^	705 ± 80^a^	661 ± 91^ad^	549 ± 84acdefg
Glucagon (pg/ml)	Milk	106 ± 5	106 ± 5	100 ± 5^ab^	93 ± 5abc	91 ± 6abc	91 ± 5abc	87 ± 5abcd	86 ± 5abcd
	Control	107 ± 5	109 ± 5	104 ± 5^b^	93 ± 4abc	91 ± 4abc	89 ± 4abc	82 ± 5abcdef	83 ± 4abcdef
GIP (pmol/l)	Milk	18 ± 3	—	—	19 ± 3	—	18 ± 2	—	—
	Control	19 ± 3	—	—	18 ± 3	—	17 ± 3	—	—

Note

GIP: gastric inhibitory peptide.

Compared with ^a^baseline, ^b^10, ^c^20, ^d^30, ^e^45, ^f^60 and ^g^90 min (*p* < 0.05).

**Figure 1:**
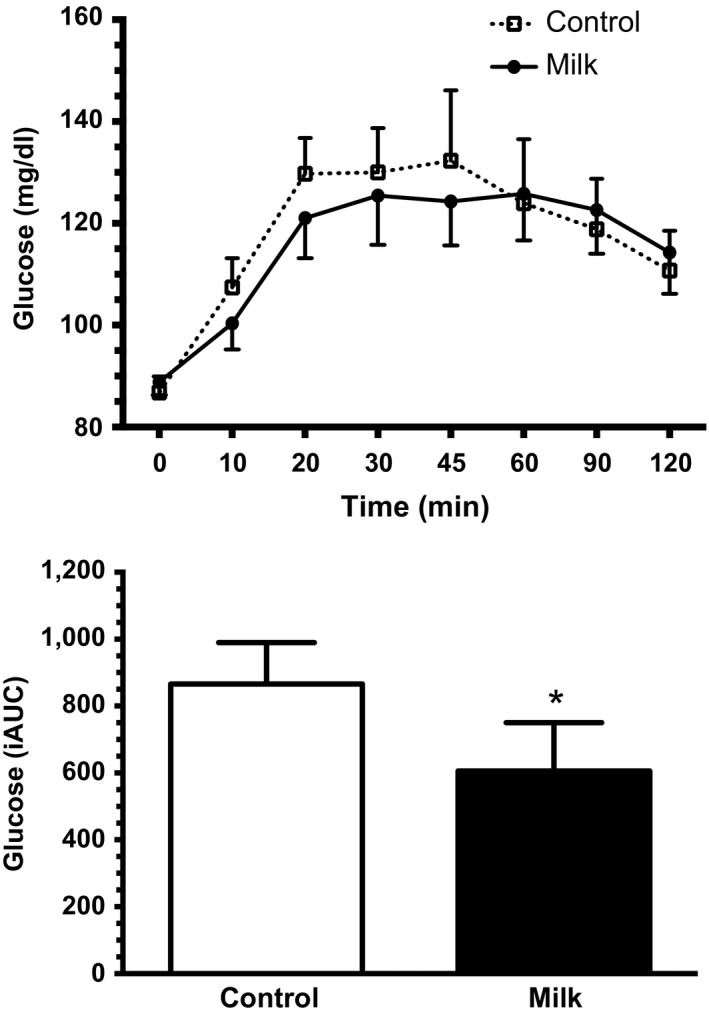
Changes in blood glucose concentration and mean glucose integrated area under the curve (iAUC) at 30 min during the OGTT with nonfat milk or the placebo control drink in the participants in the highest android body fat tertile. *compared with control drink

Relative increases in peak blood glucose concentration were significantly related to android body fat levels (*r* = 0.27, *p* < 0.05). In order to determine the influence of abdominal body fatness on metabolic responses, participants were divided into tertiles of android body fat. As expected, a greater proportion of males presented with android obesity, but there were no differences in any of the primary outcomes between sexes for the highest tertile of android obesity. The participants in the highest tertile of android body fat (>50% android body fat), comprised of 10 adults (80% men and 20% women), displayed attenuated hyperglycemic responses when supplemented with nonfat milk, compared with the placebo control beverage (Figure 1).

**Figure 2:**
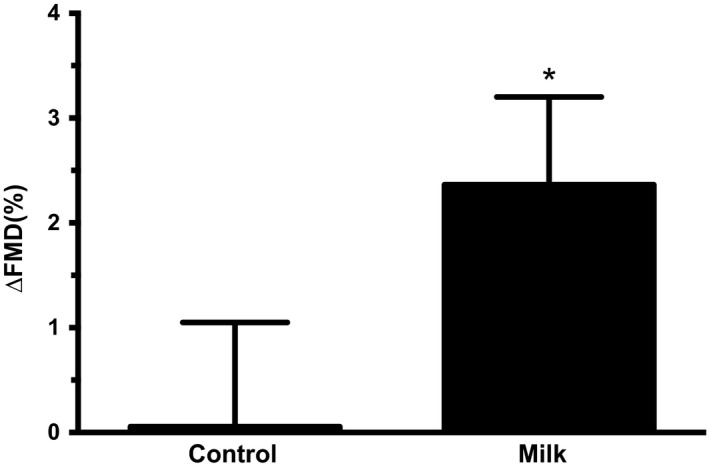
Change in flow‐mediated dilation (FMD) from baseline to the end of the OGTT (120 min) with nonfat milk or the placebo control drink in the participants in the highest android body fat tertile. *compared with control drink

Whole group vascular and hemodynamic measures are presented in Table 3. Heart rate, mean blood pressure, and brachial artery FMD did not change significantly during the observation period in both treatments. Femoral artery blood flow and vascular conductance demonstrated time effects with no differences between test beverages. In the highest tertile of android body fat group, FMD was improved (*p* < 0.05) at 120 min with the milk supplementation (Figure 2).

**Table 3:** Changes in vascular and hemodynamic measures at baseline and at the end of the oral glucose tolerance test (OGTT) with nonfat milk or the control drink

Measure	Trial	OGTT		
		Baseline	30 min	120 min
Mean blood pressure (mmHg)	Milk	84 ± 3	81 ± 2	79 ± 3
	Control	85 ± 2	83 ± 1	77 ± 4
Heart rate (bpm)	Milk	72 ± 3	70 ± 2	71 ± 2
	Control	68 ± 2	71 ± 2	71 ± 2
Brachial flow‐mediated dilation (%)	Milk	7.6 ± 0.6	8.4 ± 0.6	8.4 ± 0.6
	Control	8.7 ± 0.6	7.1 ± 0.6	8.0 ± 0.7
Femoral blood flow (ml/min)	Milk	482 ± 42	313 ± 28^a^	413 ± 45^b^
	Control	463 ± 52	310 ± 31^a^	353 ± 27^a^
Femoral vascular Conductance (AU)	Milk	5.9 ± 0.6	3.8 ± 0.4^a^	4.9 ± 0.5
	Control	5.6 ± 0.7	3.7 ± 0.4^a^	4.4 ± 0.4

Notes. Compared with ^a^baseline, ^b^30 min.

## 4 DISCUSSION

In this rigorously controlled, randomized clinical trial, the effect of nonfat milk was compared with a placebo drink matched for macronutrient and caloric content. In this study design, any differences in postprandial hyperglycemia between the nonfat milk and placebo trials could not be attributed to the macronutrient, and specifically the protein quantity, of milk. Because postprandial metabolism is affected greatly by diet and physical activity, these lifestyle factors were tightly controlled.

The salient findings of the present study were that in individuals with the highest android obesity, nonfat milk attenuated acute hyperglycemia and that the beneficial effects of dairy intake were associated with the elevated endothelium‐dependent vasodilation. These findings suggest that milk consumed with a high‐carbohydrate meal may reduce hyperglycemic responses preferentially in individuals with less favorable cardiometabolic profile and that these metabolic effects may be related to hemodynamic improvements.

In general, studies investigating the effects of specific milk proteins are more conclusive in terms of demonstrating beneficial effects on glycemic responses especially in individuals with elevated risks. For example, the consumption of whey protein with a high glycemic meal decreased postprandial serum glucose in patients with diabetes (Jakubowicz et al., 2014). We recruited obese yet young, apparently healthy participants that exhibited fairly normal postprandial metabolic and vascular responses. However, by selecting individuals with highly elevated android body fat, we observed a significant effect of milk on attenuating postprandial hyperglycemia. These results are consistent with the previous observation that the beneficial effects of dairy on metabolic and clinical parameters are more likely to be evident in individuals with elevated risk factors for CVD as there appears to be a distinct “basement effect” associated with dairy intake, in which little to no changes have been observed in healthy individuals with normal or low values (Elwood et al., 2010; Pereira et al., 2002; Poppitt et al., 2002). Therefore, the observed effects can likely be extended to, and perhaps greater in, older obese adults, type 2 diabetes, and those with CVD. Clearly, further research in these populations is warranted.

Previous studies have reported that when low‐fat milk was consumed ad libitum with a meal, postmeal blood glucose level was decreased (Panahi et al., 2013, 2013). However, because neither the foods nor beverage intakes were controlled, it remains unknown if a single serving of nonfat milk, which is typically consumed with a meal, is sufficient to induce suppression of hyperglycemia after the high‐carbohydrate intake. In the present study, we controlled and standardized the amount of carbohydrate meal as well as milk intake. Additionally, because type and amount of prior physical activity and meals could significantly affect postprandial metabolism and vascular responses, we implemented strict dietary and physical activity controls prior to each treatment. We found no significant differences in glycemic responses between the trials involving nonfat milk and those with the control drink that included the same amount of protein. These results confirm previous findings that at least some of the beneficial effects of milk on glycemic responses can be attributed to its protein quantity (Akhavan et al., 2010, 2014; Ballard et al., 2013; Elwood et al., 2010; Fekete et al., 2016; Gannon et al., 1992; Jakubowicz et al., 2014; Manders et al., 2014).

The protein component in nonfat milk is ~20% whey and ~80% casein protein. Although the total protein quantity of the control drink was identical to nonfat milk, it contained only whey protein. Whey protein is more quickly digested and absorbed than casein protein causing it to appear in the bloodstream sooner. Whey protein ingestion reduces postprandial hyperglycemia without increases in C‐peptide release or insulin concentrations suggesting that whey may affect glucose clearance by stimulating insulin‐independent mechanisms (Akhavan et al., 2010, 2014). In spite of this, we observed improvements in the early phase of the OGTT in the highest tertile of android obesity. Therefore, any insulin‐independent effects of whey do not appear to be responsible for the observed improvements in glycemic responses. While the protein quantity between the milk and control beverages was matched, the protein composition, including potential hydrolysates or amino acid content was not. Therefore, this could influence noninsulin‐dependent glycemic control.

To the best of our knowledge, this is the first study to demonstrate that milk can elicit glycemic improvements independent of the macronutrient composition, more specifically protein quantity. Importantly, previous studies investigating the effects of milk and milk protein on postprandial hyperglycemia employed less carbohydrate and more protein (Akhavan et al., 2010; Jakubowicz et al., 2014), allowing for a greater potential of protein to limit increases in blood glucose. The present findings indicate that a single serving of milk was effective in attenuating the glycemic effect of high‐carbohydrate meals at least in individuals with highly elevated android obesity. An exceptional review on the physiological effects of milk stated that future studies should examine the minimum dose at which dairy protein exerts hypoglycemic effects (Fekete et al., 2016) and to our knowledge, the present study confirmed the lowest dose yet of 8 grams of dairy protein.

Other components of milk may also be responsible for the attenuated glycemic response. One potential explanation is the presence of casein in milk. While the total protein quantity was matched, nonfat milk consists of 80% casein, whereas the macronutrient/caloric control was 100% whey. Casein is a slowly digested protein that remains in the stomach longer and delays gastric emptying. Therefore, the hypoglycemic effect during the nonfat milk trial may be mediated by a higher casein content compared with the macronutrient/caloric control trial which would have slowed the release of glucose into the small intestine and thus its absorption into the bloodstream.

Other contributors to the attenuated glycemic response could be the bioactive compounds in milk. The combined vitamin and mineral content comprises less than 1% of milk, but this rather small volume could offer significant functional properties (Rice, Cifelli, Pikosky, & Miller, 2011). Oral magnesium supplementation, for example, improves insulin sensitivity, glucose homeostasis, and HbA1c levels in diabetic patients (Bo & Pisu, 2008). Several intervention studies have shown improved glycemic responses with vitamin D treatment, but these effects may be specific to individuals who were deficient at baseline or those who had preexisting metabolic disorders (von Hurst, Stonehouse, & Coad, 2010; Rice et al., 2011). The micronutrient profile of dairy could contribute to attenuated hyperglycemia as the cellular influx of calcium plays a pivotal role in nutrient intake and in endothelial‐dependent vasodilation, and magnesium is essential for optimal coupling and signaling through the insulin receptor (Rice et al., 2011; Zemel, 2001).

Nearly, all studies showing the beneficial effects of dairy intake have attributed the favorable metabolic effects to the insulinotropic properties of milk proteins. Indeed, compared with other protein sources, milk demonstrates a larger insulin response up to 240 min postmeal (von Post‐Skagegard et al., 2006). As there were no differences in insulin responses between beverages, the effects of dairy on acute hyperglycemia in this study appear to be insulin‐independent. Throughout the intervention, there were no changes in GIP, although its sister hormone, GLP‐1, was not measured. As GLP‐1 is the more active hormone during hyperglycemia, there is the possibility that by not measuring GLP‐1, some mechanism of action contributing to the observed differences was missed, including possible differences in glucose uptake in peripheral tissues.

Our integrative physiological approach allowed us to gain insight into potential mechanisms underlying the effects of milk on postprandial metabolism. Specifically, we addressed the hypothesis that improvements in postprandial hyperglycemia would be associated with increases in peripheral perfusion and vasodilation. Compared with an isocaloric volume of rice milk, obese individuals demonstrate improved endothelial‐dependent vasodilation with low‐fat milk (Ballard et al., 2013). However, neither the meal nor the carbohydrate or protein differences were controlled between the test beverages. Additionally, whey‐derived protein elicited improved endothelium‐dependent vasodilation at 120 min in mildly hypertensive, overweight participants (Ballard et al., 2013). We also observed the beneficial effect of milk intake on FMD at the end of the OGTT, although blood glucose concentration was no longer different between the trials. One may argue that the reduced glycemic load at 30 min may have elicited a delayed effect in improved vasodilation that became evident at 120 min. Nevertheless, the improvement in FMD implies vascular protective effects of milk and significant therapeutic applications for at‐risk individuals.

The two test beverages differed not only in casein/whey content, but also in micronutrients. This is because our intention was to equate the two beverages in terms of the total calories and macronutrient quantity (i.e., total protein). As it is nearly impossible to create a control drink that exactly matches milks’ macro‐ (including protein hydrolysates and amino acids) and micronutrient profile, we recognize that this is a limitation to the present study. Other limitations include not measuring other hormonal regulators of glycemic control, such as GLP‐1, as described previously, and DPP‐4, which is partly responsible for the degradation of incretins.

Importantly, the present findings are the first to demonstrate that nonfat milk is capable of attenuating postprandial hyperglycemia independent of protein quantity in individuals with elevated android obesity. Based on the previous epidemiological studies, attenuating postprandial hyperglycemia offers tremendous potential to reduce future CV risks. The present findings indicate that a single serving of nonfat milk was sufficient to attenuate acute hyperglycemia at least in individuals with highly elevated android obesity. This offers a simple, convenient, and easily implemented option for managing elevations in blood glucose in individuals at high risk of developing CVD.

## CONFLICT OF INTEREST

The lead author affirms that this manuscript is an honest, accurate, and transparent account of the study being reported. The reporting of this work is compliant with CONSORT^1^ guidelines. The lead author affirms that no important aspects of the study have been omitted and that any discrepancies from the study as planned have been explained. This trial was registered at clinicaltrials.gov as NCT 02894112.

## AUTHOR CONTRIBUTION

MPL, DM, and HT conceived and designed the experiments. MPL, SR, JL, and WP performed the experiments. MPL, SR, RF, and JE analyzed the data. MPL and HT wrote the manuscript. All authors have read and approved the present work.

## ETHICS STATEMENT

This study conforms to the Declaration of Helsinki. All protocols and procedures were ethically reviewed and approved by the Institutional Review Board at the University of Texas at Austin. Written and verbal informed consent was collected from all participants prior to participation. The authors have no conflict of interests to report.
